# Temporal trends and variation in out-of-pocket expenditures and patient cost sharing: evidence from a Chinese national survey 2011–2015

**DOI:** 10.1186/s12939-021-01480-9

**Published:** 2021-06-19

**Authors:** Vicky Mengqi Qin, Yuting Zhang, Kee Seng Chia, Barbara McPake, Yang Zhao, Emily S. G. Hulse, Helena Legido-Quigley, John Tayu Lee

**Affiliations:** 1 Saw Swee Hock School of Public Health, National University of Singapore, Singapore, Singapore; 2 Melbourne Institute, Applied Economic & Social Research, Faculty of Business and Economics, University of Melbourne, Melbourne, Victoria Australia; 3 Nossal Institute for Global Health, School of Population and Global Health, University of Melbourne, Melbourne, Victoria Australia; 4 Centre for Health Policy, School of Population and Global Health, University of Melbourne, Melbourne, Victoria Australia; 5 Public Health Policy Evaluation Unit, Department of Primary Care and Public Health, School of Public Health, Imperial College London, London, UK

**Keywords:** User fees, Cost- sharing, Out-of-pocket payment, Geographical variation, Socioeconomic disparity, China

## Abstract

**Objectives:**

This study aims to examine: (1) temporal trends in the percentage of cost-sharing and amount of out-of-pocket expenditure (OOPE) from 2011 to 2015; (2) factors associated with cost-sharing and OOPE; and (3) the relationships between province-level economic development and cost-sharing and OOPE in China.

**Setting:**

A total of 10,316 adults aged ≥45 years from China followed-up from 2011 to 2015 were included in the analysis. We measured two main outcome variables: (1) patient cost sharing, measured by the percentage of OOPE as total healthcare expenditure, and (2) absolute amount of OOPE.

**Results:**

Based on self-reported data, we did not find substantial differences in the percentage of cost sharing, but a significant increase in the absolute amount of OOPE among the middle-aged and older Chinese between 2011 and 2015. The percentage of cost-sharing was considerably higher for outpatient than inpatient care, and the majority paid more than 80% of the total cost for prescription drugs. Provinces with higher GDP per capita tend to have lower cost-sharing and a higher OOPE than their counterparts, but the relationship for OOPE became insignificant after adjusting for individual factors.

**Conclusion:**

Reducing out-of-pocket expenditure and patient cost sharing is required to improve financial protection from illness, especially for those with those with chronic conditions and reside in less developed regions in China. Ongoing monitoring of financial protection using data from various sources is warranted.

Strengths and limitations of this study

- This is the first longitudinal study to measure the trend of and variation in patient cost-sharing and OOPE in China.
- User fees were self-reported by the respondents, which may be subject to recall bias.
- User fees in this study only reflected the general cost burden from formal health care services; therefore, user fees from informal care services were not captured.

Key findings

- There were no substantial reduction in patient cost-sharing for outpatient and inpatient services, but the amount of out-of-pocket expenditure (OOPE) continued to rise during 2011–2015.
- Despite universal health insurance coverage, patient cost-sharing was still high among the middle-aged and older Chinese: 84.0% for outpatient care and 69.2% for inpatient care in 2011; and 80.8% vs 62.2% in 2015. The majority of patients paid more than 80% of the total cost for prescription drugs when visiting outpatient or inpatient care.
- Several patient-level characteristics affected cost-sharing and OOPE, including insurance status, age, education, household economic status and number of chronic conditions. Cost-sharing was lower for those with insurance compared to those without insurance.
- Provinces with higher GDP per capita had lower cost-sharing than provinces with lower GDP per capita, but no significant difference was found in the amount of OOPE after controlling for individual-level factors.

## Introduction

Protection against catastrophic levels of health spending as a result of illness has been a key goal of health systems in many countries [1, 2]. User fees, defined as direct payment at the point of seeking care paid by patients, remain the primary source of health care financing in many low-and middle-income countries [3–6]. Access to health care is inversely related to income and socioeconomic status, where wealthier groups have better access to high-quality health care than the poorer groups [7, 8].

In China, almost the entire population (more than 95% in 2013) is covered by one of the three social health insurance schemes: the New Rural Cooperative Medical Scheme (NCMS), the Urban Resident Basic Medical Insurance (URBMI), and the Urban Employee Basic Medical Insurance (UEBMI). Insured patients’ user fees include deductibles (i.e., fees paid out-of-pocket below deductible thresholds), copayment (i.e., a certain percentage applied to the fees beyond deductible and below reimbursement ceiling), and patient payment above the reimbursement ceiling (i.e., fees beyond upper limit of copayment is paid out-of-pocket, which should be at least six times of the average income). The design of user fees vary by social health insurance scheme [1, 9, 10].

At the end of 2015, the Chinese government announced the decision to integrate URBMI and NCMS as the Urban-Rural Resident Medical Insurance Scheme. This integration has enabled a further extension of funding pools and narrowing disparities in access to health care services and medications that existed between different insurance schemes [11]. The three social health insurance schemes are designed to target different populations. The NCMS targets the registered rural population; the URBMI and UEBMI target the urban non-employed residents and employees, respectively. UEBMI generally provides more comprehensive service coverage (including both outpatient and inpatient services) and lower cost-sharing compared with the other two schemes [12]. In addition, user fees for the same health insurance scheme can vary significantly across provinces due to fiscal capacity and priority setting of local governments [13]. Official statistics reported that per capita OOPE increased more than three folds in the past two decades (2000–2019), i.e., from 85 Yuan to 290 Yuan for outpatient care, and from 3083 Yuan to 9848 Yuan for inpatient care [14].

Two recent multli-country studies of universal health coverage of over 100 countries found that China has a higher incidence of catastrophic health expenditure compared to countries with similar economic development levels [15, 16]. Low level of benefit coverage and fragmented social health insurance schemes could be the reason for the high incidence of catastrophic health expenditure, in particular among the rural, poorer and sicker population [17]. It is worth noted that the increasing demand of health care and forgone timely treatment among the rapidly aging population has also driven up OOPE [18–20]. In addition, spending on medications has become a major component of total health expenditure (41.9% in 2010) [21]. Thus, improving financial protection is crucial for health system strengthening in China, and the Chinese government has set an ambitious target to substantially reduce patient cost-sharing (i.e. the percentage of out-of-pocket payment in total health expenditure) from 60% in 2001 to 25% by 2030 [22].

The literature on the provincial level variation in user fees is relatively sparse in China [13, 23–25]. A recent cross-sectional analysis of the key parameters of different health insurance programs found that cost-sharing varies significantly by insurance schemes in China [23]. However, there is no longitudinal study to comprehensively document the individual and contextual factors associated with user fees and how user fees change over time. The answers to two research questions remain unclear: (1) How do user fees change over time in China? (2) What are factors associated with the variation in user fees in China? Understanding these questions could provide policies for social health insurance reforms in China. Using the longitudinal data from 2011 to 2015 of the China Health And Retirement Longitudinal Study (CHARLS), we examine: (1) trends in cost-sharing and OOPE among middle-aged and older adults in China; (2) socioeconomic factors associated with user fees, and (3) the relationships between province-level economic development and user fees in China.

## Methods

### Data

We used the longitudinal data from the China Health And Retirement Longitudinal Study (CHARLS) conducted in 2011, 2013, and 2015. CHARLS adopted the multi-stage stratified probability proportional to size sampling method at baseline. CHARLS had collected a nationally representative sample aged 45 years and above from 150 counties in 28 provinces. At baseline in 2011, 17,708 respondents (80.5% response rate) were interviewed, and 13,565 (76.6% of baseline sample) were followed up concurrently for three waves [26]. We identified 10,316 respondents, after removing ineligible respondents aged 45 years and below or with missing values in covariates.

### Measurements and variables

We measured user fees regarding: patient cost- sharing, defined as the ratio of out-of-pocket payment (OOPE) in total health care spending, and the actual amount of OOPE (in Chinese Yuan) [27]. We calculated them for outpatient and inpatient services separately. In addition, we also calculated user fees for prescription drugs which have been a major component of health spending in China [21]. We examined the association between socioeconomic determinants, geographic region, and user fees.

Respondents who sought outpatient care last month or inpatient care last year were asked: “*What was the total cost of this visit (or hospitalisation), including both treatment and medication cost (or fees paid to the hospital)?*”, and “*How much did you pay out of pocket, after reimbursement from insurance (for the total costs of hospitalisation)?*” Similarly, respondents were asked: “*What was the total medication cost for this visit, including prescription you received?*”, and “*How much will you eventually pay out of pocket for the medications from this visit, including prescriptions you received?*” for outpatient and inpatient settings, respectively. If there was no cost or respondents did not pay for the visits or medications, then patient cost-sharing was denoted as 0. Likewise, if the respondents further reported that the outpatient or inpatient visits were not covered by any insurance, then cost-sharing was denoted as 1.

Socioeconomic indicators that may vary user fees were included in the analysis as independent variables, including: health insurance type (UEBMI, URBMI, NCMS, others, without insurance), location (rural, urban), gender, age (45–54, 55–64, 65–74, ≥75), marital status (single/divorced/widowed, married/cohabitated), number of self-reported doctor-diagnosed NCDs at the individual level (none, 1 type of NCD, 2 types of NCDs, ≥ 3 types of NCDs), working status (working, retired, non-working), household economic status (the most deprived, deprived, middle, affluent and the most affluent), education (elementary school and below, secondary school, college and above) and time (year) [23, 28, 29]. Respondents with both social health insurance schemes and other types of health insurance (e.g. private insurance, government medical insurance (Gong Fei) and other supplementary insurance) were coded as “others”. We included 13 types of NCDs that are available in CHARLS for the calculation: hypertension, dyslipidaemia, diabetes, cancer, chronic lung disease, liver disease, heart disease, stroke, chronic kidney disease, digestive disease, mental disorders, arthritis and asthma. Household economic status was defined based on quintiles of yearly per capita household consumption. Per capita household consumption was based on a relative approach by comparing yearly per capita household consumption to the median value at the city level to reduce bias from imbalance economic development across regions [30]. We also explored the relationship between user fees and economic development. We identified and ranked economic development at the provincial level based on their GDP per capita: low, < 4300 US$; middle, 4300–12,000 US$; high, ≥12,000 US$ (1 USD = 6.2 CNY in 2014). The cut-off points for grouping provinces were referred to the country classifications from the World Bank [31].

### Statistical analysis

We measured socioeconomic and provincial inequality in user fees using a series of regression-based methods [32]. We adopted a four-level random intercept linear regression model to explore the association between socioeconomic determinants and user fees, and to control for individual heterogeneity and measure external effects. OOPE was log-transformed in regression to allow for a more intuitive interpretation of regression coefficients as percentage changes in OOPE. The multilevel model accounted for hierarchical nature of the CHARLS data, with individuals at the first level, and community, city and province at the second, third and fourth level, respectively.

Therefore, cost-sharing (denoted as Y_ijkl_) of individual i, living in community j of city k in province l in time t, given his/her sociodemographic characteristics can be described as follows:

$$ {\mathrm{Y}}_{\mathrm{ijk}\mathrm{l}}={\beta}_0+\beta \ast {\mathrm{X}}_{\mathrm{ijk}\mathrm{l}}+{\beta}_{time,i}\ast {\mathrm{t}}_{\mathrm{ijk}\mathrm{l}}+{\mathrm{v}}_{\mathrm{k}}+{\mathrm{u}}_{\mathrm{jk}}+{\mathrm{e}}_{\mathrm{ijk}}+{\mathrm{g}}_{\mathrm{ijk}\mathrm{l}} $$

$$ {\mathrm{v}}_{\mathrm{k}}\sim N\ \left( 0,{\sigma^2}_{\mathrm{v}}\right) $$

$$ {\mathrm{u}}_{\mathrm{jk}}\sim N\ \left( 0,{\sigma^2}_{\mathrm{u}}\right) $$

$$ {\mathrm{e}}_{\mathrm{ijk}}\sim N\ \left( 0,{\sigma^2}_{\mathrm{e}}\right) $$

$$ {\mathrm{g}}_{\mathrm{ijkl}}\sim N\ \left( 0,{\sigma^2}_{\mathrm{g}}\right) $$

Where Y_ijkl_ is the predicted cost- sharing, *β*_0_ is the mean cost-sharing across participants, X_ijkl_ represents the vector of all independent variables that were adjusted for in the analysis with *β as the fixed effect*, *β*_*time,i*_ counted for the time effect, v_k_, u_jk_, e_ijk_ and g_ijkl_ represent the random effect of province, city, community, and individual respectively, assuming an independent and normal distribution with zero mean and constant variances (*σ*^*2*^_v_, *σ*^*2*^_u_, *σ*^*2*^_e_, *σ*^*2*^_g_).

We also measured the variance of cost-sharing attributable to each level of the multilevel model by calculating variance partition coefficients (VPC). We excluded outliers with an extremely high value of health expenditures (i.e. > 30,000 Yuan for outpatient (0.08% among the respondents without lost-to-follow-up), before the calculation. To allow comparison over time, OOPE reported in 2011 and 2013 were converted to 2015 price using the gross domestic product (GDP) deflator according to the World Bank [33]. Adjusted coefficient (β) and 95% confidence intervals (CI) were presented for multilevel models, with *p* < 0.05 taken as statistically significant. All statistical analyses were conducted using STATA 16.0.

## Results

We analysed panel data from 10,316 respondents observed in 2011, 2013, and 2015. Table 1 summaries the sociodemographic characteristics of the respondents. At baseline, the majority of the respondents were female (51.1%), aged 55–64 years (38.9%), residing in rural areas (58.7%), currently working (71.4%), and attained elementary education or below (64.6%) in 2015. More than 65% of the respondents had at least one type of diagnosed NCD. More than 94% of participants were enrolled in at least one of the insurance schemes, with the majority insured by NCMS (73.7%).

**Table 1:** Sociodemographic characteristics of sample at baseline

	N (%)
**Health insurance**	
No insurance	602 (5.8)
NCMS	8072 (73.7)
URBMI	470 (5.5)
UEBMI	922 (12.1)
Others	250 (2.9)
**Location**	
Urban	3575 (41.3)
Rural	6741 (58.7)
**PCE**	
Lowest 20%	1170 (694)
Lower 20%	2697 (1164)
Middle 20%	4500 (1801)
Higher 20%	7548 (2797)
Highest 20%	14,260 (17328)
**Employment**	
Not working	252 (2.5)
Retired	2417 (26.1)
Working	7647 (71.4)
**Education**	
Elementary or below	6861 (64.6)
Secondary school	3314 (33.4)
College and above	141 (2)
**Age (years)**	
45–54	3921 (38.9)
55–64	4150 (38.9)
65–74	1818 (17.8)
≥ 75	427 (4.4)
**Gender**	
Male	5086 (48.9)
Female	5230 (51.1)
**Marital**	
single or divorce or widowed	955 (9.8)
married or cohabitated	9361 (90.2)
**Comorbidity**	
None	3450 (34.2)
1 NCD	3244 (31.3)
2 NCDs	1997 (18.9)
≥ 3 NCDs	1625 (15.5)
**N**	10,316

Note:

•All results are weighted to account for complex survey design.

•*Abbreviation*: *UEBMI* Urban Employee Basic Medical Insurance, *URBMI* Urban Resident Basic Medical Insurance, *NCMS* New Rural Cooperative Medical Scheme, *Others* government health care, private medical insurance and others, *NCD* Non-communicable disease, *PCE* Per capita expenditure (Chinese Yuan)

•median PCE of each wealth group was displayed with standard deviation in parentheses

### The trend in user fees 2011–2015

Overall, there was no substantial reduction in patient cost-sharing between 2011 and 2015. However, the amount of OOPE continued to rise within the 4 year period (from an average of 457 Yuan to 860 Yuan for outpatient, and from 4861Yuan to 5747 Yuan for inpatient services).

Patient cost-sharing was higher for outpatient than inpatient care (e.g. 82% vs 67% in 2011 and 80% vs 63% in 2015). The majority of the patients had to pay more than 80% of the total cost of prescription drugs when visiting outpatient or inpatient care.

Across the three major types of social health insurance schemes, participants enrolled in UEBMI had a lower cost-sharing compared with participants enrolled in URBMI and NCMS. Participants enrolled in urban insurance (UEBMI or URBMI) had a higher OOPE than those insured by NCMS in rural areas (Table 2). From 2011 to 2015, the differences in absolute amount of the OOPE across three social health insurance schemes for outpatient services narrowed, but the differences in percentage of cost sharing increased. In inpatient setting, both the differences in OOPE and cost sharing in general reduced. (Appendix Table 1).

**Table 2:** Patient Out-of-pocket Expenditure and Cost-sharing by Health Insurance schemes during 2011–2015

Insurance status	Total	None	NCMS	URBMI	UEBMI	Other	*P* value
	**2011**						
**Outpatient**							
Total cost	677 (571, 782)	567 (290, 844)	540 (442, 638)	771 (488, 1054)	1031 (663, 1399)	1731 (622, 2839)	< 0.01
OOPE for doctor visits^a^	457 (388, 525)	478 (188, 768)	431 (348, 514)	545 (322, 767)	475 (309, 642)	719 (307, 1131)	< 0.01
Cost sharing^a^	0.82 (0.79, 0.85)	0.9 (0.83, 0.98)	0.87 (0.84, 0.89)	0.7 (0.53, 0.88)	0.68 (0.58, 0.77)	0.57 (0.44, 0.7)	< 0.01
Amount of OOPE for prescription drug^b^	298 (259, 338)	387 (120, 655)	251 (221, 281)	324 (177, 471)	423 (274, 572)	473 (23, 923)	< 0.01
Cost sharing for prescription drugs^b^	0.88 (0.85, 0.91)	0.97 (0.93, 1)	0.92 (0.9, 0.94)	0.74 (0.55, 0.93)	0.78 (0.69, 0.87)	0.68 (0.54, 0.82)	< 0.01
Seek outpatient care observation	3286						
**Inpatient**							
Total cost	7971 (7008, 8935)	5550 (4073, 7027)	6903 (5699, 8108)	7494 (4878, 10,110)	11,902 (9342, 14,462)	8756 (5769, 11,742)	< 0.01
OOPE for hospitalization^a^	4861 (4245, 5477)	4150 (3107, 5193)	4732 (4081, 5382)	4816 (2845, 6786)	5706 (3659, 7754)	3658 (2123, 5193)	< 0.01
Cost sharing^a^	0.67 (0.64, 0.7)	0.91 (0.83, 1)	0.74 (0.71, 0.77)	0.67 (0.59, 0.75)	0.44 (0.39, 0.5)	0.54 (0.41, 0.67)	< 0.01
OOPE for prescription drugs^b^	3668 (3131, 4205)	2884 (1976, 3791)	3674 (3023, 4325)	4252 (2304, 6200)	3913 (2458, 5368)	2354 (1070, 3637)	< 0.01
Cost sharing for prescription drugs^b^	0.85 (0.82, 0.87)	0.9 (0.81, 0.99)	0.86 (0.84, 0.89)	0.89 (0.85, 0.94)	0.77 (0.72, 0.83)	0.8 (0.71, 0.89)	0.049
Seek inpatient care observation	1560						
	**2013**						
**Outpatient**							
Total cost	753 (647, 859)	653 (351, 955)	717 (608, 826)	888 (490, 1286)	818 (524, 1111)	825 (190, 1460)	< 0.01
OOPE for doctor visits^a^	522 (441, 602)	578 (330, 826)	548 (466, 631)	546 (236, 855)	445 (244, 647)	245 (117, 372)	< 0.01
Cost sharing^a^	0.76 (0.69, 0.83)	0.96 (0.92, 1)	0.85 (0.84, 0.87)	0.61 (0.4, 0.82)	0.51 (0.35, 0.68)	0.49 (0.36, 0.62)	< 0.01
Amount of OOPE for prescription drug ^b^	358 (305, 411)	294 (133, 455)	364 (299, 429)	418 (208, 628)	335 (237, 433)	204 (91, 317)	< 0.01
Cost sharing for prescription drugs^b^	0.86 (0.82, 0.91)	0.94 (0.85, 1.03)	0.93 (0.92, 0.94)	0.78 (0.51, 1.04)	0.67 (0.58, 0.76)	0.61 (0.48, 0.73)	< 0.01
Seek outpatient care observation	3958						
**Inpatient**							
Total cost	10,082 (7258, 12,906)	9077 (6507, 11,648)	7654 (6922, 8387)	25,659(− 1260, 52,578)	11,672 (8219, 15,124)	10,722 (7868, 13,575)	< 0.01
OOPE for hospitalization ^a^	6508 (4087, 8929)	6372 (4666, 8079)	5047 (4484, 5611)	20,955(− 3563, 45,472)	5454 (3896, 7012)	6535 (1762, 11,308)	< 0.01
Cost sharing^a^	0.62 (0.59, 0.65)	0.99 (0.97, 1.01)	0.65 (0.62, 0.67)	0.66 (0.56, 0.76)	0.48 (0.4, 0.55)	0.4 (0.29, 0.5)	< 0.01
OOPE for prescription drugs ^b^	5419 (2951, 7888)	4944 (3614, 6273)	3852 (3348, 4356)	20,367(− 4290, 45,023)	4594 (3044, 6145)	5198 (2122, 8274)	0.0037
Cost sharing for prescription drugs^b^	0.83 (0.81, 0.85)	0.99 (0.97, 1.01)	0.84 (0.82, 0.86)	0.82 (0.76, 0.87)	0.8 (0.74, 0.85)	0.79 (0.68, 0.89)	< 0.01
Seek inpatient care observation	2385						
	**2015**						
**Outpatient**							
Total cost	1279 (1076, 1482)	1744 (600, 2888)	1094 (898, 1290)	1169 (668, 1670)	1562 (1121, 2002)	2786(−9, 5580)	< 0.01
OOPE for doctor visits^a^	860 (705, 1015)	937 (430, 1444)	801 (640, 961)	842 (482, 1201)	813 (530, 1096)	2381(−388, 5150)	< 0.01
Cost sharing^a^	0.8 (0.77, 0.82)	0.89 (0.84, 0.93)	0.84 (0.82, 0.86)	0.88 (0.83, 0.93)	0.61 (0.53, 0.69)	0.51 (0.39, 0.64)	< 0.01
Amount of OOPE for prescription drug (Yuan)^b^	375 (309, 441)	350 (167, 534)	316 (264, 368)	462 (318, 607)	478 (252, 704)	797 (135, 1459)	< 0.01
Cost sharing for prescription drugs^b^	0.85 (0.83, 0.88)	0.88 (0.83, 0.93)	0.9 (0.88, 0.92)	0.91 (0.87, 0.95)	0.69 (0.61, 0.76)	0.61 (0.49, 0.73)	< 0.01
Seek outpatient care observation	3754						
**Inpatient**							
Total cost	9757 (8846, 10,668)	7207 (5633, 8782)	8194 (7487, 8901)	10,073 (8117, 12,030)	14,826 (11,850, 17,802)	8731 (5716, 11,746)	< 0.01
OOPE for hospitalization^a^	5747 (5228, 6266)	4578 (3458, 5698)	5605 (5038, 6172)	6046 (4708, 7385)	6562 (5021, 8103)	4285 (1885, 6685)	< 0.01
Cost sharing^a^	0.63 (0.61, 0.65)	0.9 (0.84, 0.96)	0.67 (0.65, 0.69)	0.59 (0.56, 0.63)	0.44 (0.4, 0.47)	0.43 (0.29, 0.56)	< 0.01
OOPE for prescription drugs ^b^	4418 (3922, 4915)	3822 (2790, 4853)	4206 (3702, 4710)	4298 (3072, 5525)	5374 (3728, 7021)	3126 (895, 5356)	< 0.01
Cost sharing for prescription drugs^b^	0.84 (0.82, 0.86)	0.93 (0.88, 0.99)	0.86 (0.84, 0.87)	0.83 (0.78, 0.88)	0.77 (0.71, 0.83)	0.75 (0.59, 0.9)	< 0.01
Seek inpatient care observation	2590						

Note:

The currency unit of total cost and OOPE in this table is Chinese Yuan.

OOPE and cost sharing were presented followed by 95% CI in the parenthsis.

These tables were generated based on the full sample of year wave so as to track the national trend.

OOPE = out-of-pocket expenditure

*P* value< 0.01 indicates that the mean level of cost-sharing was statistically different between three social health insurance schemes at 1% significance level, based on Kruskal–Wallis test.

Cross-sectional sampling weight was applied for each year.

OOPE in 2011 and 2013 were converted to 2015 price based on the World Bank GDP deflator.

^a^Indicates the total cost shared or paid by patients, including prescription drugs

^b^ Indicates only the total cost for prescription drugs shared or paid by patients

Provinces with higher GDP per capita tend to have lower cost-sharing but higher OOPE for outpatient and inpatient care, compared with provinces with lower GDP per capita. (Appendix Figure 1 & 2) Among provinces with high GDP per capita, the average cost-sharing was 73.1% (1341 Yuan) for outpatient visits, and 60.8% (7641 Yuan) for inpatient visits in 2015. In comparison, the average cost-sharing was 80.7% (579 Yuan) for outpatient visits, and 67.0% (4505 Yuan) for inpatient visits among provinces with low GDP per capita. (Data not shown in tables).

### Percentage of cost-sharing

#### Outpatient

Outpatient cost-sharing was significantly lower among the insured respondents (regression coefficient = − 0.09, − 0.09, − 0.21, − 0.28, for NCMS, URBMI, UEBMI, and other health insurance respectively, *p* < 0.05) than the uninsured counterparts (Table 3). People who were in the older age group (regression coefficient = − 0.02, − 0.05, − 0.08, for those aged 55–64, 65–74, and 75 and above, *p* < 0.05), retired (regression coefficient = − 0.09, *p* < 0.05), and had tertiary education (regression coefficient = − 0.16, p < 0.05) had lower cost-sharing compared with those aged 45–54 years old, unemployed, and primary education or below, respectively. Outpatient cost-sharing was lower among respondents from regions with high GDP per capita compared to respondents from regions with low GDP per capita (regression coefficient = − 0.09, *p* < 0.05). Outpatient cost-sharing was not associated with gender, marital status, household economic status, and number of NCDs. There was no significant change in outpatient cost-sharing during 2011 and 2015 (*p* > 0.05).

**Table 3:** Determinants of patient cost-sharing for outpatient and inpatient services from multilevel regression analysis

	Outpatient				Inpatient			
	Overall		Medicines only		Overall		Medicines only	
	Regression coefficient	*P* value	Regression coefficient	*P* value	Regression coefficient	*P* value	Regression coefficient	*P* value
**Insurance type (ref: no insurance)**								
NCMS	− 0.089 (− 0.127, − 0.051)	< 0.001	− 0.054(− 0.086, − 0.023)	0.001	− 0.281(− 0.334, − 0.229)	< 0.001	− 0.111(− 0.164, − 0.058)	< 0.001
URBMI	− 0.094 (− 0.144, − 0.043)	< 0.001	− 0.063(− 0.105, − 0.02)	0.004	− 0.316(− 0.382, − 0.25)	< 0.001	− 0.134(− 0.2, − 0.067)	< 0.001
UEBMI	−0.205 (− 0.252, − 0.158)	< 0.001	−0.171(− 0.211, − 0.131)	< 0.001	−0.453(− 0.513, − 0.393)	< 0.001	− 0.172(− 0.233, − 0.111)	< 0.001
Others	−0.282 (− 0.352, − 0.211)	< 0.001	−0.278(− 0.34, − 0.215)	< 0.001	−0.508(− 0.598, − 0.418)	< 0.001	−0.167(− 0.259, − 0.076)	< 0.001
**PCE (ref: lowest 20%)**								
Lower 20%	− 0.022 (− 0.048, 0.004)	0.094	0.003(− 0.019, 0.025)	0.804	0.004(− 0.036, 0.044)	0.840	− 0.025(− 0.065, 0.015)	0.221
Middle 20%	−0.025 (− 0.05, 0)	0.053	− 0.015(− 0.036, 0.007)	0.185	− 0.036(− 0.074, 0.003)	0.071	− 0.021(− 0.06, 0.018)	0.297
Higher 20%	−0.015 (− 0.041, 0.01)	0.236	− 0.002(− 0.023, 0.02)	0.885	0.002(− 0.035, 0.039)	0.929	− 0.026(− 0.064, 0.011)	0.166
Highest 20%	− 0.02 (− 0.046, 0.006)	0.128	− 0.007(− 0.029, 0.015)	0.540	0.016(− 0.02, 0.053)	0.382	−0.055(− 0.092, − 0.018)	0.004
**Employment status (ref: not working)**								
Retired	−0.091 (− 0.177, − 0.005)	0.038	−0.045(− 0.118, 0.028)	0.228	0.056(− 0.037, 0.15)	0.236	−0.033(− 0.128, 0.062)	0.495
Working	−0.064 (− 0.15, 0.021)	0.140	−0.026(− 0.099, 0.047)	0.488	0.046(− 0.048, 0.14)	0.335	−0.031(− 0.127, 0.064)	0.518
**Education (ref: primary school or below)**								
Secondary school	−0.009 (− 0.03, 0.011)	0.369	−0.004(− 0.021, 0.013)	0.652	−0.028(− 0.055, − 0.002)	0.036	0.002(− 0.024, 0.029)	0.866
College and above	−0.164 (− 0.24, − 0.089)	< 0.001	−0.155(− 0.221, − 0.089)	< 0.001	0.005(− 0.086, 0.096)	0.913	−0.104(− 0.197, − 0.012)	0.027
**Age (ref:** **45–54 years)**								
55–64	− 0.025 (− 0.045, − 0.005)	0.013	−0.013(− 0.03, 0.004)	0.125	− 0.031(− 0.061, − 0.002)	0.035	−0.012(− 0.042, 0.017)	0.411
65–74	− 0.054 (− 0.078, − 0.03)	< 0.001	−0.037(− 0.058, − 0.017)	< 0.001	−0.053(− 0.086, − 0.02)	0.002	−0.028(− 0.061, 0.006)	0.105
75 and above	−0.078 (− 0.117, − 0.04)	< 0.001	−0.074(− 0.107, − 0.04)	< 0.001	−0.082(− 0.126, − 0.037)	< 0.001	−0.053(− 0.099, − 0.008)	0.022
**Gender (ref: male)**								
Female	0.008 (−0.008, 0.025)	0.324	0.019 (0.004, 0.033)	0.010	0.031 (0.009, 0.053)	0.006	0.031 (0.009, 0.053)	0.007
**Marital (ref: single or divorce or widowed)**								
Married or cohabitated	0.004 (−0.019, 0.027)	0.716	−0.018(− 0.037, 0.002)	0.076	0.038 (0.008, 0.069)	0.014	0.004(− 0.027, 0.035)	0.784
**Comorbidity (ref: none)**								
1 NCD	0.004 (−0.023, 0.031)	0.763	0.005(−0.019, 0.028)	0.694	0.031(−0.01, 0.071)	0.138	−0.004(− 0.045, 0.037)	0.851
2 NCDs	0 (−0.027, 0.027)	0.995	0.011(− 0.013, 0.034)	0.364	0.015(− 0.024, 0.054)	0.452	−0.008(− 0.048, 0.032)	0.682
> 2 NCDs	−0.005 (− 0.032, 0.021)	0.702	0(− 0.023, 0.022)	0.976	0.007(− 0.03, 0.044)	0.696	− 0.014(− 0.051, 0.023)	0.464
**Location (ref: urban)**								
Rural	0.013 (−0.011, 0.038)	0.290	0.003(− 0.017, 0.022)	0.774	0.045 (0.016, 0.073)	0.002	−0.018(− 0.046, 0.01)	0.210
**GDP per capita (ref: low)**								
Middle	−0.015 (− 0.043, 0.013)	0.295	− 0.017(− 0.04, 0.007)	0.162	0.029(− 0.007, 0.065)	0.117	− 0.006(− 0.04, 0.028)	0.713
High	−0.09 (− 0.161, − 0.019)	0.013	−0.057(− 0.114, 0)	0.050	0.064(− 0.018, 0.145)	0.128	− 0.002(− 0.081, 0.076)	0.953
**Year (ref: 2011)**								
2013	−0.019 (− 0.041, 0.003)	0.092	− 0.002(− 0.021, 0.017)		− 0.09(− 0.122, − 0.059)	< 0.001	0.013(− 0.018, 0.044)	0.399
2015	− 0.011 (− 0.035, 0.013)	0.372	− 0.018(− 0.039, 0.002)		− 0.079(− 0.112, − 0.046)	< 0.001	0.031(− 0.001, 0.064)	0.060
**VPC, %**								
Province	1.5%		0.6%		0.0%		0.0%	
City	1.6%		1.6%		2.7%		0.5%	
Community	2.9%		1.4%		0.7%		1.8%	
Individual	94.0%		96.3%		96.6%		97.7%	

Note:

95% CI was displayed in parentheses after the regression coefficient

Respondents categorised in other insurance group were enrolled in insurance program other than the three major social health insurance, such as private insurance, government-funded insurance (Gong Fei) etc

*Abbreviation*: *UEBMI* Urban Employee Basic Medical Insurance, *URBMI* Urban Resident Basic Medical Insurance, *NCMS* New Rural Cooperative Medical Scheme, *Others* government health care, private medical insurance and others, *PCE* Per capita expenditure (Chinese Yuan), *NCD* Non-communicable disease, *VPC* Variance partition coefficient

A likelihood ratio test was conducted to compare the fully adjusted model with null model with only random intercept and multivariable linear model. Fully adjusted multilevel model is preferred (*P* < 0.01)

Likewise, people with insurance, tertiary education and older age also had lower cost-sharing of prescription drugs in outpatient setting compared to their counterparts without insurance, low education level, and aged 45–54 years. Respondents from regions with high GDP per capita (regression coefficient = − 0.05, *p* = 0.05) had significantly lower cost-sharing of prescription drugs than those from regions with low GDP per capita. Other sociodemographic covariates such as household economic status, employment status, and number of NCDs were not associated with outpatient cost-sharing of prescription drugs (*p* > 0.05).

#### Inpatient

Inpatient cost-sharing was significantly lower among respondents with health insurance (regression coefficient = − 0.28, − 0.32, − 0.45, − 0.51, for NCMS, URBMI, UEBMI, and other health insurance respectively, *p* < 0.05). Respondents who were female (regression coefficient = 0.03, p < 0.05), married (regression coefficient = 0.04, *p* < 0.05), and resided in rural area (regression coefficient = 0.05, *p* < 0.05) had higher level of cost-sharing than their counterparts. Respondents who were aged between 55 and 64, 65–74, and 75 and older had a lower level of cost-sharing (regression coefficient = − 0.03, − 0.05, − 0.08, p < 0.05), compared with those aged 45–54 years. The level of inpatient cost-sharing was lower in the year 2013 (regression coefficient = − 0.10, p < 0.05) and 2015 (regression coefficient = − 0.09, p < 0.05), compared with the year 2011. Cost-sharing for inpatient services was not significantly associated with education level, employment status, household economic status, number of NCDs, and regional economic development.

Similarly, inpatient cost-sharing of prescription drugs was lower among people with insurance, aged 75 and above, had tertiary education compared to those without insurance, aged 45–54, and had primary education or below, respectively. Inpatient cost-sharing of prescription drugs was not significantly different with regard to employment status, household economic status, location and provincial economic development (*p* > 0.05).

### Amount of OOPE

#### Outpatient

Table 4 shows that the amount of OOPE for outpatient visits was lower among respondents insured by UEBMI (regression coefficient = − 0.34, *p* < 0.05) and “other insurance” including private and government-funded insurance (regression coefficient = − 1.17, p < 0.05) compared to those without insurance. Older age groups (regression coefficient = − 0.13, − 0.21 and − 0.48 for age group 55–64, 65–74 and 75 and above respectively, *p* < 0.05) and tertiary education (regression coefficient = − 0.80, *p* < 0.05) was associated with less OOPE, compared to people aged 45–54 years and who had primary education or below respectively. Respondents from households with the most affluent economic status spent more on outpatient OOPE compared to those from the worst economic status (regression coefficient = 0.39, *p* < 0.05). Respondents who were married (coefficient = 0.21, p < 0.05) and had more NCDs (coefficient = 0.27, 0.42, 0.46, for people had two and more than two types of NCDs, p < 0.05) spent higher OOPE than their counterparts who were male, single, and without diagnosed NCDs respectively. OOPE was higher in year 2013 (regression coefficient = 0.21, *p* < 0.05) and 2015 (regression coefficient = 0.45, p < 0.05), compared with year 2011. Outpatient OOPE were also not associated with employment status, education, location, and regional economic development.

**Table 4:** Determinants of the amount of OOPE for outpatient and inpatient services from multilevel regression analysis

	Outpatient				Inpatient			
	Log of OOPE		Log of OOPE for Prescription drugs		Log of OOPE		Log of OOPE for Prescription drugs	
	Regression coefficient	*P* value	Regression coefficient	*P* value	Regression coefficient	*P* value	Regression coefficient	*P* value
**Insurance type (ref: no insurance)**								
NCMS	− 0.094(− 0.339, 0.152)	0.455	− 0.071(− 0.295, 0.152)	0.532	− 0.08(− 0.427, 0.267)	0.652	− 0.065(− 0.443, 0.314)	0.738
URBMI	0.012(− 0.312, 0.337)	0.941	0.111(− 0.186, 0.408)	0.465	− 0.021(− 0.454, 0.412)	0.925	− 0.065(− 0.538, 0.408)	0.788
UEBMI	− 0.338(− 0.642, − 0.035)	0.029	− 0.266(− 0.544, 0.011)	0.060	− 0.002(− 0.401, 0.398)	0.993	0.035(− 0.4, 0.469)	0.876
Others	−1.173(− 1.626, − 0.72)	< 0.001	−1.333(− 1.761, − 0.905)	< 0.001	−1.101(− 1.686, − 0.516)	< 0.001	− 0.347(− 0.991, 0.298)	0.292
**PCE (ref: lowest 20%)**								
Lower 20%	−0.141(− 0.306, 0.024)	0.093	− 0.048(− 0.199, 0.104)	0.538	0.115(− 0.14, 0.371)	0.376	−0.085(− 0.366, 0.197)	0.556
Middle 20%	0.092(−0.07, 0.254)	0.264	0.081(−0.067, 0.23)	0.283	0.182(− 0.066, 0.43)	0.151	0.097(−0.177, 0.372)	0.486
Higher 20%	0.112(−0.051, 0.275)	0.178	0.131(−0.018, 0.28)	0.085	0.493 (0.255, 0.732)	< 0.001	0.222(−0.04, 0.484)	0.097
Highest 20%	0.391 (0.226, 0.556)	< 0.001	0.361 (0.21, 0.512)	< 0.001	0.759 (0.523, 0.995)	< 0.001	0.326 (0.066, 0.586)	0.014
**Employment status (ref: not working)**								
Retired	−0.156(− 0.712, 0.4)	0.582	−0.102(− 0.611, 0.408)	0.696	0.202(− 0.402, 0.806)	0.513	− 0.466(−1.127, 0.195)	0.167
Working	− 0.444(− 0.998, 0.109)	0.116	−0.407(− 0.915, 0.1)	0.116	−0.158(− 0.765, 0.448)	0.608	−0.739(− 1.401, − 0.077)	0.029
**Education (ref: primary school or below)**								
Secondary school	− 0.001(− 0.13, 0.128)	0.988	0.011(− 0.107, 0.129)	0.859	−0.096(− 0.271, 0.079)	0.282	0.071(− 0.115, 0.257)	0.456
College and above	−0.802(− 1.275, − 0.328)	0.001	−0.488(− 0.941, − 0.036)	0.035	−0.056(− 0.654, 0.541)	0.853	−0.78(− 1.42, − 0.14)	0.017
**Age (ref:** **45–54 years)**								
55–64	− 0.13(− 0.257, − 0.002)	0.046	−0.108(− 0.224, 0.008)	0.069	− 0.163(− 0.354, 0.027)	0.093	−0.162(− 0.368, 0.043)	0.121
65–74	− 0.208(− 0.361, − 0.056)	0.008	−0.145(− 0.285, − 0.005)	0.042	−0.425(− 0.64, − 0.21)	< 0.001	−0.356(− 0.588, − 0.124)	0.003
75 and above	−0.482(− 0.727, − 0.237)	< 0.001	−0.381(− 0.608, − 0.154)	0.001	−0.74(− 1.033, − 0.447)	< 0.001	−0.498(− 0.814, − 0.181)	0.002
**Gender (ref: male)**								
Female	0.036(−0.071, 0.143)	0.508	0.021(−0.077, 0.119)	0.680	−0.129(− 0.273, 0.016)	0.081	−0.106(− 0.261, 0.048)	0.177
**Marital (ref: single or divorce or widowed)**								
Married or cohabitated	0.212 (0.066, 0.357)	0.004	0.075(−0.059, 0.209)	0.273	0.482 (0.283, 0.681)	< 0.001	0.145(−0.069, 0.359)	0.185
**Comorbidity (ref: none)**								
1 NCD	0.268 (0.095, 0.44)	0.002	0.273 (0.115, 0.432)	0.001	0.159(−0.103, 0.421)	0.233	0.114(−0.172, 0.4)	0.435
2 NCDs	0.417 (0.243, 0.59)	< 0.001	0.425 (0.265, 0.585)	< 0.001	0.071(−0.184, 0.326)	0.584	−0.005(− 0.283, 0.272)	0.971
> 2 NCDs	0.46 (0.291, 0.628)	< 0.001	0.431 (0.276, 0.586)	< 0.001	0.129(−0.111, 0.369)	0.291	0.021(−0.24, 0.281)	0.877
**Location (ref: urban)**								
Rural	0.091(−0.061, 0.243)	0.240	0.049(−0.084, 0.183)	0.469	0.001(−0.184, 0.187)	0.988	−0.349(− 0.554, − 0.144)	0.001
**GDP per capita (ref: low)**								
Middle	−0.131(− 0.312, 0.05)	0.157	− 0.104(− 0.273, 0.064)	0.225	0.236(− 0.004, 0.476)	0.054	0.146(− 0.103, 0.395)	0.251
High	− 0.208(− 0.669, 0.254)	0.378	−0.064(− 0.498, 0.371)	0.773	0.649 (0.094, 1.204)	0.022	0.605 (0.011, 1.2)	0.046
Year (ref: 2011)								
2013	0.212 (0.07, 0.354)	0.003	0.137 (0.006, 0.269)	0.041	−0.096(−0.297, 0.104)	0.347	0.049(−0.174, 0.272)	0.664
2015	0.447 (0.292, 0.602)	< 0.001	0.155 (0.011, 0.299)	0.035	−0.039(− 0.252, 0.175)	0.723	0.136(− 0.1, 0.371)	0.258
**VPC, %**								
Province	2.0%		3.0%		1.1%		0.3%	
City	2.3%		2.4%		0.6%		0.0%	
Community	1.8%		1.1%		1.9%		3.8%	
Individual	93.9%		93.5%		96.4%		95.9%	

Note:

95% CI was displayed in parentheses after the regression coefficient

Respondents categorised in other insurance group were enrolled in insurance program other than the three major social health insurance, such as private insurance, government-funded insurance (Gong Fei) etc

*Abbreviation*: *UEBMI* Urban Employee Basic Medical Insurance, *URBMI* Urban Resident Basic Medical Insurance, *NCMS* New Rural Cooperative Medical Scheme, *Others* government health care, private medical insurance and others, *OOPE* Out-of-pocket expenditure, *PCE* Per capita expenditure (Chinese Yuan), *VPC* Variance partition coefficient

A likelihood ratio test was conducted to compare the fully adjusted model with null model with only random intercept and multivariable linear model. Fully adjusted multilevel model is preferred (*P* < 0.01)

OOPE was log-transformed to normalise the distribution. Patients with zero OOPE were replaced by 1 for a mathematically meaningful log-transformation

Outpatient OOPE of prescription drugs were also lower among respondents insured by UEBMI and “other insurance”, being in older age group groups than the uninsured group and those aged 45–54 years. Respondents from the most affluent households (coefficient = 0.36, *p* < 0.05) and who had more NCDs (coefficient = 0.43, for people who had two and more than two types of NCDs, *p* < 0.05) spent a higher amount of OOPE compared their counterparts. No significant difference was found for outpatient OOPE of prescription drugs with regards to gender, marital status, employment status, location, and provincial economic development.

#### Inpatient

OOPE for inpatient services did not significantly differ by the type of social health insurance compared to those without any insurance. However, people covered by “other insurance” such as private insurance (regression coefficient = − 1.10, *p* < 0.05) spent less OOPE than those without insurance. People in the older age groups spent less for inpatient OOPE compared to those aged 45–54 years (regression coefficient = − 0.43, − 0.74, for 65–74 and 75 and above, respectively, *p* < 0.05). Respondents who were married (regression coefficient = 0.48, *p* < 0.05) and had the most affluent household economic status (regression coefficient = 0.76, *p* < 0.05) spent more on inpatient OOPE compared to those single and with the most deprived household economic status. Respondents from provinces with middle and high GDP per capita spent more on OOPE (regression coefficient = 0.24, 0.65, for middle and high GDP per capita respectively *p* < 0.05) compared to those from provinces with low GDP per capita. Inpatient OOPE was not associated with gender, employment status, education, number of NCDs, and location.

Respondents who were in the older age group (regression coefficient = − 0.35, − 0.49, for aged 65–74 and 75 and above respectively, *p* < 0.05), employed (regression coefficient = − 0.74, p < 0.05), and had tertiary education (regression coefficient = − 0.80, *p* < 0.05) spent less on OOPE for prescription drugs during hospitalisation, compared to those aged 45–54 years and unemployed, respectively. People from the most affluent household (regression coefficient = 0.33, p < 0.05) spent more on OOPE for prescription drugs than their counterparts from the most deprived household. OOPE for prescription drugs was significantly higher among respondents from regions with high GDP per capita compared to respondents from regions with low GDP per capita (regression coefficient = 0.61, p < 0.05). Inpatient OOPE for prescription drugs was not associated with gender, the number of NCDs, and location.

#### Partitioning variations in user fees

In the fully adjusted model for cost- sharing, 1.5% of the variation in outpatient cost-sharing comes from provinces, 1.6% from cities, 2.9% from communities within cities, and 94% lies within the community between individuals (Table 3). In inpatient settings, individuals accounted for 96.6% of the variation in cost-sharing, followed by 0.7 and 2.7% at the community and city-level respectively.

Variation in outpatient OOPE was similar, with individuals accounted for 93.9% of the variation, followed by communities (1.8%), cities (2.3%) and provinces (2%). Individual-level accounted for 96.4% of the variation in inpatient OOPE, with community, city and province-level accounted for 1.9, 0.6 and 1.1% (Table 4).

## Discussion

### Principal findings

Findings from the longitudinal dataset of 10,316 respondents aged 45 years and above in China, revealed no substantial reduction in the percentage of cost-sharing over time, but the amount of OOPE continued to rise. Cost-sharing and OOPE were lower among those insured than the uninsured group, with UEBMI than the other social health insurance schemes, and with private insurance than the social health insurance schemes. Provinces with higher GDP per capita in Beijing, Shanghai and Tianjin tend to have lower cost-sharing but higher OOPE than those provinces with lower GDP per capita in Yunnan, Guizhou, Gansu.

Several studies have examined level of user charges in China using different types of dataset, including administrative dataset and survey data. While our results are comparable with previous studies that were based on CHARLS dataset [34, 35], our findings on level of cost-sharing might be higher than other studies that reported ‘reimbursement rate’ in China [36]. Unlike reimbursement rate, which generally refers to the percentage of medical costs covered by health insurance for health services within the coverage of health insurance, our measurement of cost-sharing also include patient’s out-of-pocket expenditure for health services that are outside of service coveage of the social health insurance programmes. It is why our estimates for levels of cost-sharing are not “identical” to the reimbursement- rate reported in other studies.

Our findings showing patient cost-sharing was higher for the outpatient than inpatient visits, reflecting the better coverage for inpatient services in China. We found that people from households with higher economic status incurred a higher amount of OOPE from seeking treatment for their illness, which is similar to the conclusion from previous cross-sectional studies [23, 25]. This is likely because those households with higher economic status have a higher demand of health care use and tend to visit higher-tiers of health care providers (such as seondary and tertiary hospitals) [37].

There are several important caveats of our study. Firstly, self-reported user fees in surveys may not be as accurate as health insurance claim data due to the expenditure categories used and the recall period. This limitation could led to higher recall bias and lower validity of the data compared to the administrative data [38, 39]. It is possible that levels of cost sharing or amount of OOPE estimated from this study will be different from those estimated based on different types of dataset such as administrative data [38]. Secondly, user fees in this study measured the general cost burden for seeking health care. Therefore, user fees for specific types of disease were not available. Thirdly, data on expenditure was only available among adults who visited outpatient care last month or inpatient care last year. It is possible that those who did not seek care have better health conditions and face different user fees. Generalisability of the results might also be exclusive of people aged 45 years and above who may have different patterns and determinants of user fees. Lastly, our findings of user fees only reflect the level of financial protection from formal health care services of hospital and clinics. Therefore, user fees from informal care (such as purchasing medicines over the counter) were not captured and beyond the scope of this study [40, 41].

### Policy implications

One of the key focuses of current health financing reform in China is to reduce user fees, but few studies have examined this. We found that the percentage of cost-sharing for prescription drugs remained high nationwide. The high cost-sharing for outpatient raises concern for an aging population who need long-term access to outpatient care and medication treatment. Recent evidence shows that the introduction of a reimbursement for the outpatient cost of NCDs in NCMS has not reduced the incidence of catastrophic health expenditure effectively [42]. It is fundamental to further extend social health insurance to cover more health services. Targeted intervention to lower cost-sharing for long-term and chronic prescription drugs for people with NCDs is needed to enhance financial support of social health insurance [43]. Although the national policy reform of essential medicines reduced drug expenditure at inpatient settings, it has yet to significantly reduce drug expenditure at outpatient settings and for total health spending [44, 45]. Policies to broaden the benefits package of social health insurance, such as expanding the essential medicines list, should be prioritised [46]. While policies to lower mark-up for prescription drugs can reduce price, complementary financing mechanism such as government subsidies should be considered to counteract the income loss suffered by health care facilities and providers from drug sales [21]. Effectively lowering cost-sharing is an essential strategy to make the needed health services accessible and affordable, that could reduce inequalities and yield more substantial and sustainable impacts on overall population health [47, 48]. Although a few provinces have benefited from relatively low cost- sharing, high cost-sharing (higher than 30%, a threshold considered as low level of financial protection) and increasing OOPE in most provinces, especially people from rural regions, should not be neglected [12].

Reducing patient user fees by expanding government subsidies alone is not sufficient to improve financial protection and access. It is also important to improve quality and efficiency of the healthcare system; that is, to reduce low-value care and promote high-value low cost medical care including primary care and preventive care [49, 50]. The current health insurance schemes in China reimburse mainly on hospital care but not on outpatient primary care. In addition, the hospital-centric delivery system does not encourage the use of primary care [36, 51, 52]. China should continue to invest in strengthening primary care if it were to shift towards primary care-based demand for health care. This includes building primary care infrastructure, recruiting and training more quality primary care professionals, and educate the public on the value of preventive care services as a gate-keeper of health.

Future research is needed not only on examining the effect of reducing user fees on health outcomes, and different health benefits gained from user fees reduction from different types of health care, but also explore whether user fees inequality could mitigate with additional improvement in the process of healthcare delivery, such as quality and efficiency of primary care, and a shift to primary care-based health delivery system [43].

## Conclusion

Despite near universal health insurance coverage, variation in user charges across proviences and population groups persist. A complementary reform of the fragmented social health insurance schemes and the integrated healthcare delivery system are required to improve financial protection and mitigate inequality from illness for vulnerable groups such as those with chronic conditions, especially in less developed regions in China. Ongoing monitoring of user fees using various source of datasets including national survey and administrative dataset is required to guide policy making at national and sub-national levels.

## Figures and Tables

**Fig. 1:**
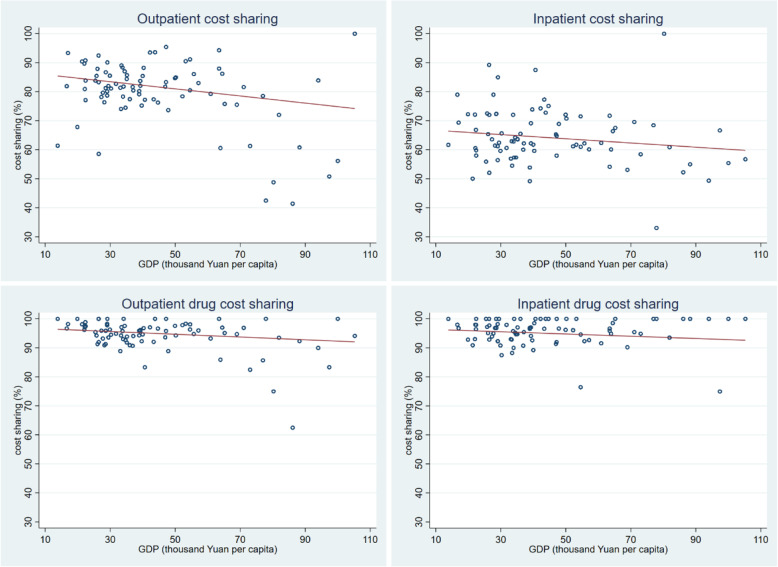
Bivariate relationship between cost-sharing and economic development at the provincial level between 2011 and 2015 (*n* = 84). Note: Provincial GDP per capita data were extracted from China Statistical Yearbook 2016. Each province (*n* = 28) has three independent data points

**Fig. 2:**
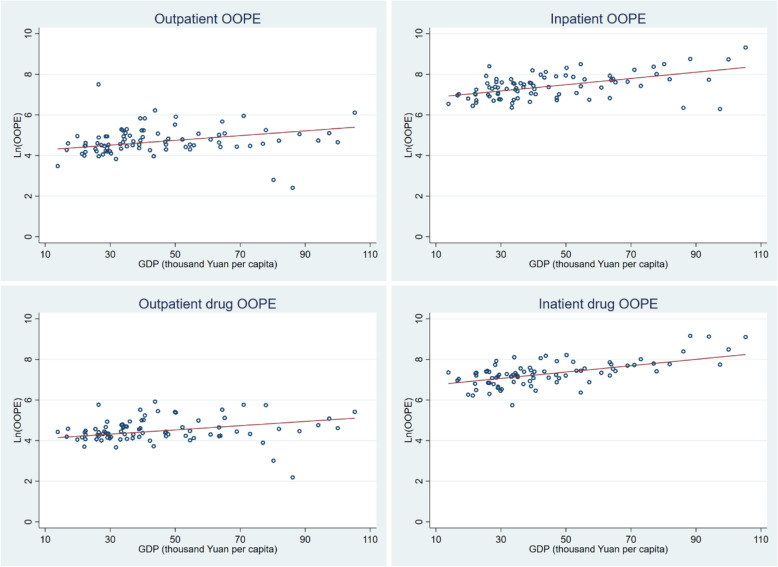
Bivariate relationship between OOPE and economic development at the provincial level between 2011 and 2015 (*n* = 84). Note: Provincial GDP per capita data was extracted from China Statistical Yearbook 2016. OOPE was log-transformed to normalise the distribution. OOPE in 2011 and 2013 were converted to 2015 price-based GDP deflator. Each province (n = 28) has three observations

## Data Availability

The datasets supporting the conclusions of this article are publicly available in the CHARLS website: http://charls.pku.edu.cn/index.html

## Appendix

**Table 5:** Comparison of out-of-pocket expenditure and cost sharing across social health insurance schemes

	UEBMI	Differece with URBMI	Differece with NCMS	Differece with non-insured
2011				
Outpatient OOPE (Yuan)	475	70	−44	3
Outpatient cost sharing (%)	68	2	19	22
Inpaitent OOPE (Yuan)	5706	− 890	− 974	− 1556
Inpatient cost sharing (%)	44	23	30	47
2013				
Outpatient OOPE (Yuan)	445	101	103	133
Outpatient cost sharing (%)	51	10	34	45
Inpaitent OOPE (Yuan)	5454	15,501	− 407	918
Inpatient cost sharing (%)	48	18	17	51
2015				
Outpatient OOPE (Yuan)	813	29	−12	124
Outpatient cost sharing (%)	61	27	23	28
Inpaitent OOPE (Yuan)	6562	− 516	− 957	− 1984
Inpatient cost sharing (%)	44	15	23	46

*OOPE* Out-of-pocket expenditure, *UEBMI* Urban Employee Basic Medical Insurance, *URBMI* Urban Resident Basic Medical Insurance, *NCMS* New Rural Cooperative Medical Scheme

## Abbreviations

GDP: Gross domestic product
NCD: Non-communicable diseases
NCMS: New cooperative medical scheme
OOPE: Out-of-pocket payment
UEBMI: Urban employee basic medical insurance
URBMI: Urban resident basic medical insurance
